# Polysaccharides from Sea Cucumber (*Stichopus japonicus*) Synergize with Anti-PD1 Immunotherapy to Reduce MC-38 Tumor Burden in Mice Through Shaping the Gut Microbiome

**DOI:** 10.3390/foods14030387

**Published:** 2025-01-24

**Authors:** Jiahui Li, Jinhui Jia, Yue Teng, Xiaojuan Wang, Xiaojun Xia, Shuang Song, Beiwei Zhu, Xiaodong Xia

**Affiliations:** 1 State Key Laboratory of Marine Food Processing and Safety Control, National Engineering Research Center of Seafood, School of Food Science and Technology, Dalian Polytechnic University, Dalian 116034, China; lijh@xy.dlpu.edu.cn (J.L.); jjh19980527@163.com (J.J.); foodsciyueteng@163.com (Y.T.); songs1008@163.com (S.S.); zhubeiwei@163.com (B.Z.); 2 Dalian Jinshiwan Laboratory, 1 Qinggongyuan Road, Ganjingzi District, Dalian 116034, China; 3 State Key Laboratory of Oncology in South China, Guangdong Provincial Clinical Research Center for Cancer, Sun Yat-sen University Cancer Center, Guangzhou 510060, China; wangxiaoj1@sysucc.org.cn (X.W.); xiaxj@sysucc.org.cn (X.X.)

**Keywords:** sea cucumber, polysaccharides, immune checkpoint inhibitors, gut microbiota, tumor

## Abstract

Immune checkpoint inhibitors (ICIs) have revolutionized cancer treatment and significantly improved outcomes for patients with certain malignancies. However, immunotherapy with ICIs is only effective in a subset of patients and the gut microbiota have been identified as an important factor associated with response to ICI therapy. Polysaccharides from sea cucumber (*Stichopus japonicus*) (SCP) have been shown to modulate the gut microbiota and exhibit beneficial health functions, but whether SCP could synergize with anti-PD1 immunotherapy remains unexplored. In this study, mice with ICI-sensitive MC38 tumors were treated with anti-PD1 antibody after supplementation with or without SCP to examine the potential impact of SCP on the efficacy of immunotherapy. SCP strongly amplified the anti-tumor activity of anti-PD1 in MC38 tumor-bearing mice. Flow cytometry and immunohistological staining demonstrated that SCP treatment increased cytotoxic CD8^+^ T lymphocytes while decreasing regulatory Foxp3^+^ CD4^+^ T lymphocytes. Gut microbiota and metabolomic analysis revealed that SCP modulated the microbiota and increased the abundance of certain metabolites such as indole-3-carboxylic acid. Furthermore, fecal microbiota transplantation experiments justified that the synergistic effect of SCP with anti-PD1 was partially mediated through the gut microbiota. Mice receiving microbiota from SCP-treated mice showed a boosted response to anti-PD1, along with enhanced anti-tumor immunity. These findings indicate that SCP could be utilized as a dietary strategy combined with anti-PD1 therapy to achieve improved outcomes in patients.

## 1. Introduction

The advent of immunotherapy has drastically improved the treatment outcomes of patients with different types of tumors [1]. The use of immune checkpoint inhibitors (ICIs), which target programmed cell death 1, programmed cell death-ligand 1, and cytotoxic T lymphocyte-associated protein 4 (CTLA4), has achieved success in a number of malignancies ranging from non-small-cell lung cancer, bladder cancer, renal cancer, and melanoma [2]. Nevertheless, the response rates varied in patients even with the same type of tumor [3]. Extensive efforts have been undertaken to discover the underlying mechanisms behind the relatively low response in certain patients, and a number of factors have been identified to be associated with ICI efficacy, such as baseline PD-L1 expression in tumor cells, host genetics, and the profile of tumor-infiltrating lymphocytes [4].

Among various factors impacting the anti-tumor activity of ICI therapy, the gut microbiota have been increasingly reported to be a key player in determining the clinical outcomes of patients [5]. Early epidemiology studies have revealed differences in microbiota composition between responsive and non-responsive patients [6], while recent clinical trials using fecal microbiota transplantation (FMT) consolidate the connection between the gut microbiota and ICI responsiveness [7]. Meanwhile, recent research efforts have uncovered some potential mechanisms by which the gut microbiota could influence immunotherapy. Certain microbiota members could directly interact with immune cells locally or produce metabolites that may modulate systemic anti-tumor immune response [8]. Based on this understanding of the role of microbiota in modulating ICI response, researchers have realized that manipulating the gut microbiota could be leveraged as an adjuvant strategy to improve the performance of ICI therapy [9].

Human gut microbiota contain trillions of microbes including bacteria, fungi, and viruses, and its composition could be altered in response to internal and external factors, such as exercise, antibiotics, and diet [10]. Diet could induce a substantial change in the gut microbiota, with various dietary components causing specific changes in the gut microbial ecosystem. Among those dietary substances, polysaccharides from plants or animals have been extensively explored for their beneficial effects on the gut microbiome and health [11]. Natural polysaccharides are well known for their immunomodulatory activity as well as their capacity to modify the gut microbiota [12]. Certain polysaccharides could interact directly with innate and adaptive immune cells and enhance anti-tumor activity. For example, *Astragalus* polysaccharide can polarize macrophages, promote the maturation of dendritic cells, and enhance the anti-cancer function of T cells [13,14]. Moreover, most polysaccharides cannot be digested and absorbed in the small intestine; instead, they reach the colon, where they are efficiently utilized by gut microbes, potentially leading to a better response to ICI therapy.

Sea cucumber is commonly considered a health-promoting seafood, and one of its active components is the polysaccharides it contains [15]. The polysaccharides in sea cucumbers are mainly fucosylated chondroitin sulfate (FCS) and fucoidan sulfate (FS). It has been shown that FCS from *Stichopus japonicus* contains a main chain of (→4)-β-D-GlcA-(1→3)-β-D-GalNAc-(1→) with GalNAc4S6S:GalNAc4S in a ratio of 1.5:1, and has three types of sulfated fucosyl branches that attach to C-3 of GlcA [16]. The backbone of FS is primarily composed of (→1)-Fuc(2S) (1→3)-Fuc(1→4)-Fuc(1→4)-Fuc(1→3)-Fuc(2S)(3→) [17]. They have been shown to beneficially modulate microbiota and alleviate certain inflammation- and immune-related disorders [18]. In recent years, sea cucumber polysaccharides have shown significant potential in regulating intestinal flora, improving intestinal health, and enhancing immune function. Studies have shown that dietary supplementation of sea cucumber polysaccharides improves gut health by increasing the diversity of the gut microbiota, promoting the growth of short-chain fatty acid (SCFA)-producing bacteria and sulfide-degrading bacteria while inhibiting the proliferation of harmful bacteria [19]. Another study found that sea cucumber polysaccharides affect mesenteric T lymphocyte differentiation by regulating intestinal flora and its metabolites, thereby improving allergic symptoms and intestinal mucosal damage in mice [20]. In addition, *Holothuria leucospilota* polysaccharides have also been reported to directly enhance immune function by regulating the gut flora [21]. These results indicate that sea cucumber polysaccharides play important roles in regulating intestinal flora and immune function, highlighting their potential for health promotion and disease prevention.

Although the immunomodulatory effects of polysaccharides have been documented, there remains a significant gap in our understanding of how polysaccharides from sea cucumber influence the gut microbiota and enhance the efficacy of cancer immunotherapies. The aim of this study is to investigate the potential synergistic effects of sea cucumber polysaccharides (SCPs) on anti-PD1 immunotherapy in MC38 tumor-bearing mice. Specifically, we seek to explore how SCPs influence the gut microbiota composition and whether these microbial changes contribute to enhanced immune responses and improved efficacy of anti-PD1 therapy. By elucidating the interaction between dietary polysaccharides, the gut microbiota, and cancer immunotherapy, this study provides valuable insights into how dietary interventions can be strategically utilized to optimize immunotherapeutic outcomes in cancer treatment. Additionally, this research could lay the foundation for the development of novel dietary strategies or adjuvant therapies aimed at improving the effectiveness of immunotherapy, potentially benefiting a wide range of cancer patients.

## 2. Materials and Methods

### 2.1. Preparation of SCPs

SCPs were prepared as previously described with minor modifications [22]. Briefly, the dried sea cucumbers were powdered and suspended in 1.5 L of 0.1 M sodium acetate buffer (pH 5.7) containing 3 g of papain, 5 mM of EDTA, and 5 mM of cysteine and incubated at 60 °C for 24 h. Then, the mixture was centrifuged at 5000 rpm for 20 min and the supernatant was obtained. The sulfated polysaccharides were precipitated with cetylpyridine chloride to obtain sea cucumber polysaccharides (SCPs).

### 2.2. Cell Culture

MC38 cells were purchased from the American Tissue Culture Collection and cultured in DMEM (C11995500BT, Invitrogen, Shangahi, China) supplemented with 10% fetal bovine serum and 100 mg/mL penicillin/streptomycin. Cells were grown in an incubator with a humidified atmosphere of 5% CO_2_ at 37 °C. Cells used in this study were confirmed negative for mycoplasma.

### 2.3. Mouse Experiments

Male C57BL/6 mice (8 weeks old) were purchased from Beijing Vital River Laboratory Animal Technology Co., LTD. (Beijing, China). The animal experiment was approved by the Animal Experiment Ethics Committee of Sun Yat-sen University Cancer Center (approval number: 2022000536).

SCP intervention: One week after domestication, mice were randomly divided into four groups: a control group (PBS treatment), anti-PD-1 group (anti-PD-1 monoclonal antibody (α PD-1 mAb) (BE0273, BioxCell, Beijing, China) 100 µg/animal), SCP group (200 mg/kg), and SCP + anti-PD-1 group. Eight mice were assigned to each group. The number of mice was chosen on the basis of past experience with the models and of previous reports [14,23], and the minimum number of mice necessary to achieve statistical purpose was used for ethical reasons. Mice were pretreated with or without SCPs for 10 days. Then, approximately 1 × 10^6^ MC38 cells were inoculated subcutaneously on the dorsal ventral side of the mice. After 8 days of tumor cell inoculation, αPD-1 monoclonal antibody was injected into tumor cells. The SCP group and SCP+αPD-1 group were supplemented daily with SCPs, and tumor size and weight were measured.

### 2.4. Flow Cytometry

At the end of the experiment, tumors were collected and placed in a Petri dish containing 10 mL of PBS (with 2% FBS). The tissue was washed with PBS, and the supernatant was discarded. Then, 3–5 mL of 0.25% pancreatic enzyme was added, and the tissue was gently triturated. The dish was incubated in a 37 °C water bath and oscillated for approximately 1 h to facilitate digestion. After digestion, the sample was centrifuged at 2000 rpm for 5 min at room temperature, and the supernatant was discarded. The cell pellet was resuspended in 3–5 mL of 1 × PBS medium, transferred to a cell strainer placed in a sterile Petri dish, and ground using the plunger of a 3 mL syringe. The cells were washed twice with 1 × PBS and centrifuged at 2000 rpm for 5 min at room temperature. The supernatant was discarded, and the cell pellet was resuspended in 3–5 mL of pre-cooled 1 × PBS. The resuspended cells were filtered into a 50 mL centrifuge tube using a 70 µm cell sieve and centrifuged again at 2000 rpm for 5 min. The supernatant was discarded, and 2 mL of pre-cooled 1 × PBS was added to resuspend the cells. The cell suspension was placed on ice before counting. For flow cytometry analysis, the cells were resuspended in 300 µL of flow cytometry buffer, and the number of cells was adjusted to 1 × 10^6^. Fluorescently labeled antibodies, including CD4 (47-0041-82, eBioscience), CD8 (25-0081-82, eBioscience), granzyme B (48-8898-82, eBioscience), TNF-α (17-7321-82, eBioscience), Foxp3 (560401, BD), and IFN-γ (25-7311-82, eBioscience), were each added at 1 µL. The mixture was gently mixed and incubated in the dark at 4 °C for 30 min. After incubation, the cells were washed again with PBS to remove unbound antibodies. Finally, the cells were resuspended in flow cytometry buffer for analysis using a Fortessa X-20 flow cytometer to evaluate cell surface markers.

### 2.5. Immunohistochemical Staining

Fresh tumor tissue and colon samples were preserved in 4% paraformaldehyde (PFA) at a 1:10 tissue-to-fixative ratio. Paraffin-embedded sections (5 µm thick) were stained with hematoxylin and eosin (H&E), and the stained slides were observed and photographed using a Nikon microscope. The number of immune cells (CD4^+^ and CD8^+^) was quantified using immunohistochemistry. To block endogenous peroxidase activity, the tissue was incubated with an endogenous peroxidase blocker for 10 min at room temperature. After incubating the sections with primary antibodies (anti-CD4 (25229S, Cell Signaling Technology, Shangahi, China) and anti-CD8 (98941S, Cell Signaling Technology), followed by secondary antibodies, changes in CD4^+^ and CD8^+^ T lymphocytes were analyzed and viewed using a Leica optical microscope (Leica Biosystems Imaging, Wetzlar, Germany).

### 2.6. Fecal DNA Extraction and 16S rRNA Sequencing

Mouse feces were collected and stored at −80 °C for future use in DNA extraction and analysis. Sequencing was performed by Magigene Technology (Guangzhou, China). A fecal sample of 0.25 g from each mouse was extracted by ALFA-SEQ Advanced Soil DNA Kit (mCHIP, Guangzhou, China), separately. Full-length bacterial 16S rRNA genes were generated by PCR amplification using 27F (5′-AGRGTTYGATYMTGGCTCAG-3′) and 1492R (5′-RGYTACCTTGTTACGACTT-3′) as primers with barcodes distinguishing different samples. Then, the length and concentration of the PCR products were detected by 1% agarose gel electrophoresis. PCR products were mixed into aliquots according to the GeneTools Analysis Software (Version4.03.05.0, SynGene, Boston, MA, USA). Then, the mixture of PCR products was purified with HiPure Gel Pure DNA Mini Kit (Mgbio, Shanghai, China). SMRTbell libraries were prepared from the PCR product with the SMRTbell Express Template Prep Kit 2.0 according to the manufacturer’s instructions (Pacific Biosciences, Menlo Park, CA, USA). The SMRTbell adaptors were ligated onto the purified amplicons, and the libraries from pooled and barcoded samples were sequenced on a PacBio Sequel II platform (Guangzhou Magigene Technologies Co., Ltd., Guangzhou, China) using a Sequel II binding kit 2.1. The obtained raw reads were processed by SMRT Link software version 6.0 (Pacific Biosciences), which provided circular consensus sequence (CCS) reads with high accuracy from the raw long sequence read containing multiple reads of the 16S rRNA gene sequence. Then, the CCS reads were subjected to data splitting, sequence error correction, sequence format conversion, and finally the clean data were obtained.

### 2.7. Fecal Microbiota Transplantation

The fecal microbiota transplantation experiment was based on a previous experimental method [24]. Briefly, fresh feces from donors of different groups (control, SCP) were homogenized separately. The homogenate was then resuspended in sterile saline (0.5 mL) within an anaerobic chamber. The solution was thoroughly mixed and centrifuged at 500× *g* for 5 min (4 °C). The supernatant was collected and centrifuged at 12,000 rpm for 5 min. Sterile saline was added to the sediment at a ratio of 1:3, and the sediment was resuspended, centrifuged, and collected. Part of the precipitate was used to verify the flora extraction rate, and the other part was used for FMT. Bacterial suspensions were prepared 30 min before FMT to prevent significant changes in the flora in vitro. After 7 days of antibiotic cocktail treatment (ampicillin (1 g/L), neomycin (500 mg/L), metronidazole (1 g/L), and vancomycin (1 g/L)) by gavage, recipient mice were gavaged with 200 µL of bacterial suspensions prepared from the fecal material of SCP-treated mice and control mice. Mice were divided into 4 groups: FMT control group, FMT Control+ anti-PD-1 group, FMT SCP group, and FMT SCP+ anti-PD-1 group. Mice in the FMT control group (*n* = 5) and FMT Control+ anti-PD-1 group (*n* = 5) received microbiota from donor control mice, while the FMT SCP group (*n* = 5) and FMT SCP+ anti-PD-1 group (*n* = 5) received microbiota from donor SCP mice. Tumor cells were inoculated on day 7, and αPD-1 monoclonal antibody was injected on days 11, 15, and 19 after inoculation. Tumor cells were inoculated 7 days after FMT administration, and αPD-1 monoclonal antibody was injected three times on different days after tumor inoculation.

### 2.8. Non-Targeted Metabolomic Analysis of Feces

Non-targeted metabolomics experiments were conducted following established research protocols [14]. Briefly, fresh fecal samples from mice were collected at the conclusion of the intervention and immediately stored at −80 °C. An amount of 50 mg of fecal sample was taken and homogenized with 500 μL of solution (acetonitrile/methanol/water = 2:2:1) in an ice water bath. It was then vortexed for 30 s. The sample was stored at −20 °C for 4 h, followed by centrifugation at 12,000 rpm for 12 min at 4 °C. After centrifugation, the sample was incubated in the ice water bath and vortexed for 30 s, and then further centrifuged at 12,000 rpm for 12 min at 4 °C. The supernatant was transferred into a sample vial with a micro-insert for further analysis. Additionally, 20 µL of supernatant was pooled from each sample to create a quality control (QC) sample. A QC sample was inserted into the analytical sequence at intervals of every 5–15 samples to assess the stability of the entire analysis process. The analytical platform used for this LC-MS analysis was the UHPLC system (Vanquish, Thermo Fisher Scientific, Shanghai, China) with a UPLC BEH Amide column (2.1 mm × 100 mm, 1.7 µm) coupled to Orbitrap Exploris 120 (Orbitrap MS, Thermo Fisher Scientific, Shanghai, China). The chromatographic mobile phase consisted of 95% water + 5% acetonitrile (containing 0.1% formic acid) as mobile phase A, and 47.5% acetonitrile + 47.5% isopropyl alcohol + 5% water (containing 0.1% formic acid) as mobile phase B. The flow rate was set at 0.40 mL/min, the injection volume was 10 µL, and the column temperature was maintained at 40 °C. The Orbitrap Exploris 120 mass spectrometer was used to collect primary and secondary mass spectrometry data with the Xcalibur software (version 4.4, Thermo). The detailed parameters for MS were as follows: sheath gas flow rate of 50 Arb, auxiliary gas flow rate of 15 Arb, capillary temperature of 320 °C, spray voltage of 3.8 kV (positive) or −3.4 kV (negative). The raw data were converted to the mzXML format using ProteoWizard and processed with an in-house program, which was developed using R and based on XCMS. Second mass spectrum data were subjected to peak identification, peak extraction, and peak alignment and integration. The data were then matched with an in-house MS2 database including the KEGG database, the human metabolome database, and CAS for substance annotation and the cutoff for annotation was set to 0.3. The relative abundance of metabolites was calculated (peak area of each metabolite divided by that of internal standard) and compared between the groups.

### 2.9. Statistical Analysis

Statistical analysis was performed using Graph Pad8.0 software, and differences between groups were analyzed using one-way analysis of variance (ANOVA) and a post hoc Tukey test. *p* < 0.05 was used to determine statistical significance. Data were expressed as mean ± SEM (* *p* < 0.05, ** *p* < 0.01, *** *p* < 0.00 1, **** *p* < 0.0001).

## 3. Results

### 3.1. SCPs Augmented Anti-Tumor Efficacy of Anti-PD1 in Syngeneic Mouse Tumor Models

**Figure 1:**
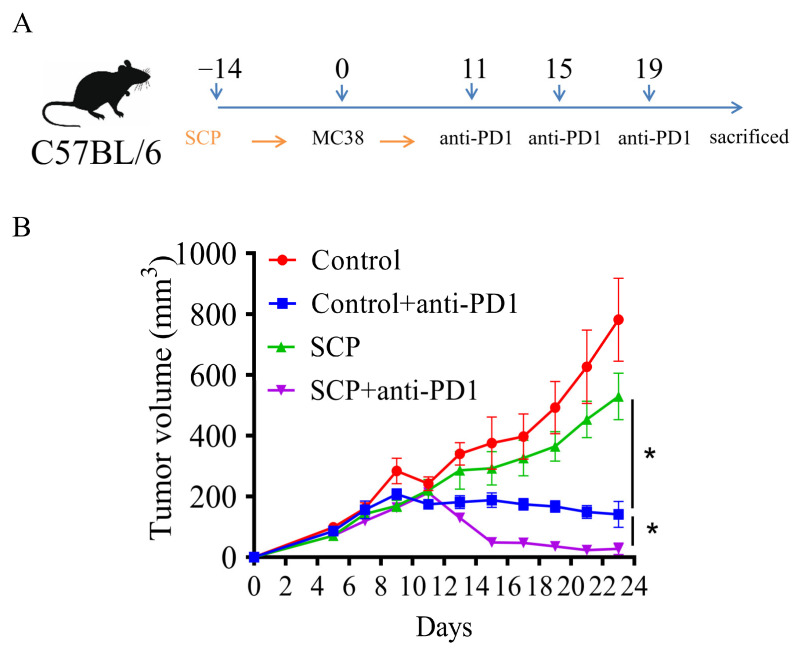
SCP-sensitized mice with CT-26 tumors to anti-PD-1 immunotherapy. (**A**) Schematic diagram of the experiment. (**B**) Tumor growth curves are shown for each group, *n* = 8 per group. * *p* < 0.05.

To investigate whether SCPs could enhance the anti-tumor effects of anti-PD1, ICI-sensitive MC-38 cells were injected into C57BL/6 mice after being supplemented with SCPs for ten days. The mice were then treated with anti-PD1 three times. SCPs alone resulted in a 32.3% reduction in tumor volume. Treatment with anti-PD1 significantly showed the growth of MC-38 tumors, showing an 81.9% reduction compared to the control group. When SCPs were combined with anti-PD1, there was a further decrease (30.6-% reduction) in tumor growth (Figure 1B), suggesting that SCPs could synergize with anti-PD1 to reduce the tumor burden in mice with MC-38 tumors.

### 3.2. SCPs Modulated Intratumor T Lymphocyte Profiles

**Figure 2:**
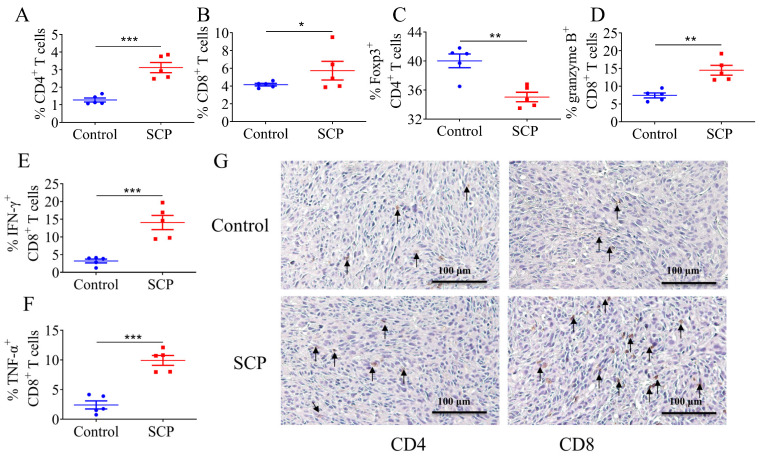
Flow cytometric and immunohistochemical (IHC) analysis of T lymphocytes in the tumors. (**A**–**F**) CD4^+^ T cell ratio, CD8^+^ T cell ratio, percentage of FOXP3^+^ CD4^+^ T, granzyme B^+^ CD8^+^ T, IFN-γ^+^ CD8^+^ T, and TNF-α^+^ CD8^+^ T cells in the tumor tissues. (**G**) Representative IHC profiles of CD4^+^ and CD8^+^ cells in tumor tissues at ×400 magnification. Arrows indicate cells positively stained with with CD4 or CD8 antibodies. *n* = 5, * *p* < 0.05, ** *p* < 0.01, *** *p* < 0.001.

To determine the impact of SCPs on immune cells in the tumor microenvironment, we analyzed the changes in CD4^+^ and CD8^+^ T lymphocytes in tumors of mice in the control and SCP groups using flow cytometry. Compared to the control group, SCPs significantly increased the levels of CD4^+^ T cells and CD8^+^ T cells (1.85% and 1.58%) (Figure 2A,B). Additionally, a notable reduction (4.98%) in Foxp3 regulatory T cells was observed in the SCP group, while the percentages of T cells expressing granzyme B (7.07%), IFN-γ (10.85%), and TNF-α (7.5%) were significantly increased (Figure 2C–F). Immunohistochemistry analysis also showed more infiltrating CD8 T^+^ cells in tumors of mice treated with SCPs, but there was no significant difference in CD4-stained cells between the two groups (Figure 2G). These results suggest that SCP treatment can enhance effector CD8^+^ T cells and suppress regulatory T cells, creating a favorable immune profile for anti-PD1 treatment.

### 3.3. SCPs Increased Microbiota Diversity and Beneficially Modified the Gut Microbiome Composition

**Figure 3:**
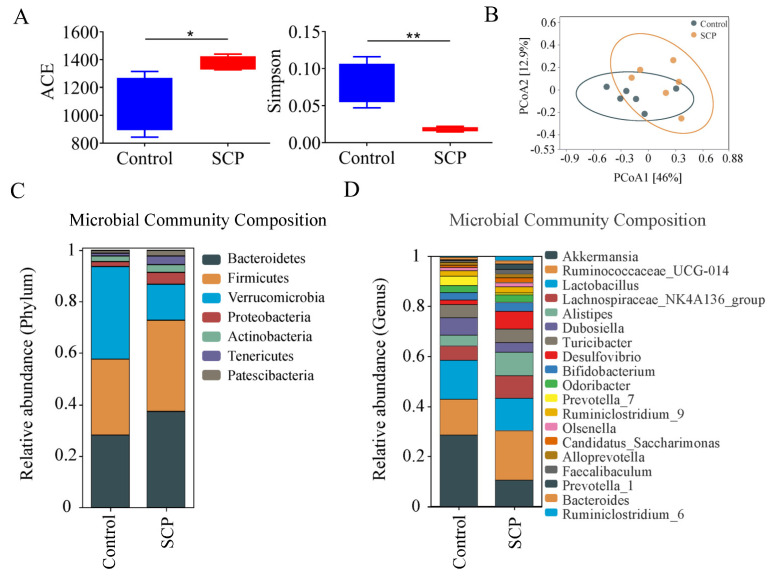
SCP treatment modulated gut microbiota in mice. (**A**) ACE and Simpson index of gut microbiome in mice treated with or without SCPs for 14 days. (**B**) Principal coordinate analysis (PcoA) of beta diversity. (**C**) Relative abundance of phylum level. (**D**) Relative abundance of species level, *n* = 6, * *p* < 0.05, ** *p* < 0.01.

Microbial profiling determined using 16S rRNA sequencing revealed that SCPs caused an increase in microbial diversity, as evidenced by a higher Abundance-based Coverage Estimator (ACE) index and a lower Simpson index. The ACE index is commonly used to measure species richness, while the Simpson index is used to measure species diversity (Figure 3A). Analysis at the phylum level showed that compared to the control group, Bacteroidetes, Firmicutes, and Proteobacteria were upregulated in the SCP group, while the abundance of Verrucomicrobia was significantly decreased (Figure 3C). At the species level, SCPs significantly enriched *Lactobacillus gasseri*, *Turicibacter* sp., *Lactobacillus reuteri*, *Bifidobacterium pseudolongum*, and *Faecalibaculum rodentium* (Figure 3D).

### 3.4. Gut Microbiota Mediated the Potentiating Function of SCPs on Anti-PD1 Treatment

**Figure 4:**
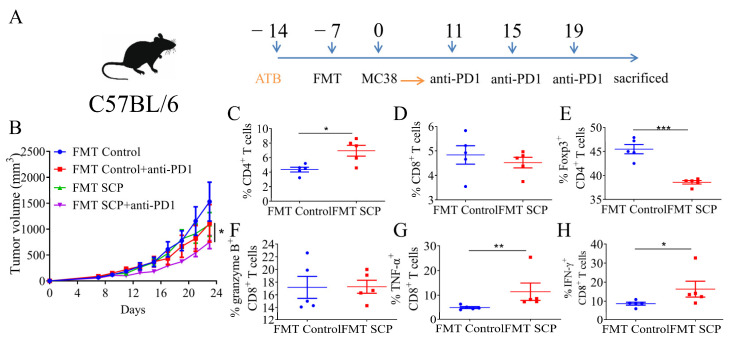
Gut microbiota mediated the potentiating effect of SCPs on the αPD-1 mAb in CT-26-tumor-bearing mice. (**A**) Schematic diagram of FMT experiment. (**B**) Tumor growth curves are shown for each group. (**C**–**H**) CD4^+^ T cell ratio, CD8^+^ T cell ratio, percentage of FOXP3^+^ CD4^+^ T, granzyme B^+^ CD8^+^ T, TNF-α^+^ CD8^+^ T, and IFN-γ^+^ CD8^+^ T cells in the tumor tissues, *n* = 5, * *p* < 0.05, ** *p* < 0.01, *** *p* < 0.001.

To test the hypothesis that the gut microbiota plays an essential role in mediating the beneficial effect of SCPs, a fecal microbiota transplantation experiment was performed. Compared with the FMT control group, the microbiota from SCPs caused a moderate reduction in tumor growth (from 1569.43 mm^3^ to 1090.86 mm^3^), which was similar to the effect of anti-PD1 antibodies (1112.25 mm^3^) in the FMT control plus anti-PD1 group. Compared to the FMT control+anti-PD1 group, the anti-tumor activity of anti-PD1 was intensively activated in the FMT SCP+anti-PD1 group, resulting in a tumor size reduction from 1112.25 mm^3^ to 750.34 mm^3^ (Figure 4B). These findings indicate that the potentiating effect of SCPs is microbiota-dependent. The microbiota from SCP-treated mice also induced changes in T lymphocytes in tumors. Compared to mice in the FMT control group, tumors of mice in the FMT SCP group contained more CD4^+^ T cells. There was a pronounced reduction in Foxp3 CD4^+^ T cells in the FMT SCP group. Although FMT-SCP treatment did not cause a significant increase in total CD8^+^ T cells, microbiota from the SCP group demonstrated an increased percentage of effector T cells, such as TNF-α^+^ CD8^+^ and IFN-γ^+^ CD8^+^ T cells (Figure 4C–H).

### 3.5. SCPs Caused Changes in Fecal Metabolomic Landscape

**Figure 5:**
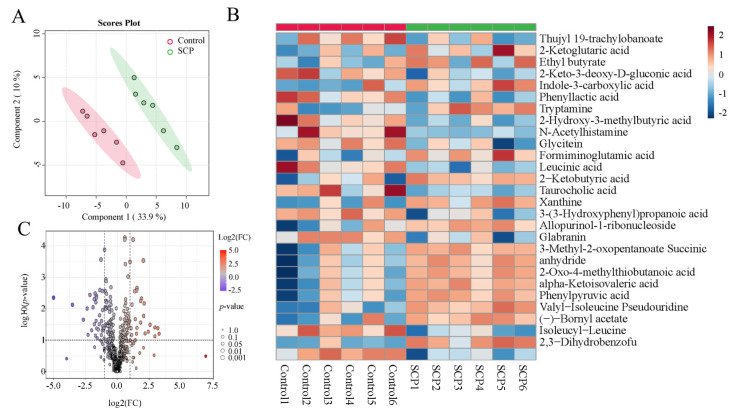
SCPs caused alterations in fecal metabolites. (**A**) PcoA of metabolites in fecal samples. (**B**) Volcano plot showing metabolites with different abundances between two groups. (**C**) Heatmap showing the differentially abundant metabolites (top 25), *n* = 6.

**Figure 6:**
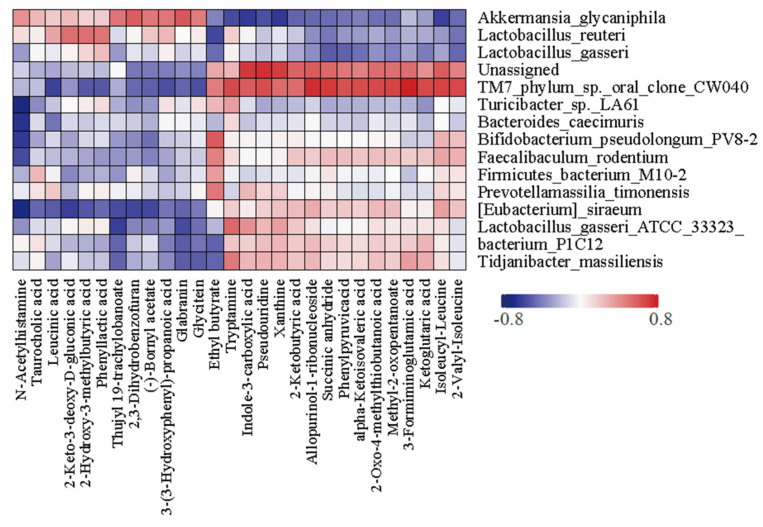
Spearman analysis of bacteria at species level and differential metabolites.

To determine whether the boosting effect of SCPs is associated with changes in some metabolites, we further investigated whether SCPs led to fluctuations in fecal metabolites. Principal coordinate analysis (PCoA) showed that metabolites in the SCP group differed from those in the control group (Figure 5A). The volcano plot in Figure 5 indicates that 33 metabolites were upregulated and 25 metabolites were downregulated in SCP-treated mice (Figure 5B). Heatmap analysis further illustrated the top 28 metabolites with significant changes (VIP > 1), and significantly upregulated metabolites such as indole-3-carboxylic acid in the SCP group were clearly visible (Figure 5C). Spearman correlation analysis suggested that certain bacterial genera are linked to specific metabolites. For example, *Lactobacillus gasseri* show a positive correlation with indole-3-carboxylic acid (Figure 6). These findings demonstrate that SCPs can induce changes in specific metabolites, potentially contributing to their positive effect on the efficacy of anti-PD1.

## 4. Discussion

Immune checkpoint inhibitors (ICIs) such as PD1, PDL1, and CTLA-4 have revolutionized cancer treatment and have benefited patients with different types of cancers. However, the relatively low response rate and immune-related adverse events hinder their broad application in clinical settings. For instance, even for the most well-known PD-1 antibody approved by the FDA, only 20 percent of patients showed a response, while the remaining patients gained no or limited benefits from anti-PD1 treatment [25]. Strategies to enhance the efficacy of ICIs are urgently needed. There is increasing evidence to support that the gut microbiota serve as a key determinant of patients’ response to ICI therapy [26]. Various diet components could modify the composition and function of the gut microbiome, influencing the response to ICIs [27]. In this study, we demonstrated that SCPs from sea cucumber synergized with anti-PD1 to reduce tumor growth in MC-38 tumor-bearing mice. The improved response was associated with favorable changes in tumor-infiltrating T cells and the gut microbiome. We further demonstrated the role of microbiota in mediating the synergistic effects of SCPs through fecal microbiota transplantation. Metabolomic analysis revealed that SCPs enriched certain metabolites known to improve ICI efficacy.

In recent years, polysaccharides have gained significant attention as natural active ingredients in anti-tumor drug research due to their remarkable anti-tumor effects. For example, polysaccharides derived from *Rosa roxburghii* have been shown in zebrafish models to exert anti-tumor effects by activating the immune system and inhibiting angiogenesis, highlighting their potential as therapeutic agents in cancer treatment [28]. Similarly, our study demonstrated that the combination of sea cucumber polysaccharides (SCPs) with anti-PD-1 therapy significantly reduced the tumor size in a colon cancer mouse model compared to the effects of anti-PD-1 treatment alone, suggesting a synergistic effect between SCPs and ICIs in enhancing anti-tumor immunity. Several studies have focused on altering the tumor microenvironment to enhance anti-tumor immunity. Fucoidan has been shown to reduce the percentage of CD3^+^ FoxP3^+^ Tregs while increasing the proportion of CD4^+^ and CD8^+^ T cells [29], providing experimental evidence for the immune enhancement induced by polysaccharides. These changes in immune cell populations are crucial as they shift the immune response towards a more robust anti-tumor profile. *Astragalus* polysaccharides inhibit PD-L1 and decrease the stem cell features of melanoma cells, which overcomes the immune evasion of tumor cells and enhances the function of immune cells [30]. In this study, SCPs increased the percentage of T cells expressing cytotoxic molecules such as granzyme B, TNF, and IFN-γ. The increase in granzyme B, one of the key molecules in cell killing action, and TNF and IFN-γ, which are potent immune activators, implies that the efficacy of T cells against tumor cells is significantly enhanced. This is reminiscent of studies in which polysaccharides from *Ganoderma lucidum* elevate the proportion of cytotoxic CD8^+^ T cells and Th1 helper cells reduce immunosuppressive Tregs, leading to activated anti-tumor immunity and enhanced responses to anti-PD1 in colorectal cancer models [31]. T cell stemness indicates the capacity of T cells for self-renewal, multipotency, and functional persistence, and it could also be critical for ICIs to work efficiently. Lactate could increase the stem-like CD8^+^ T cell population in MC38 tumors, potentiating anti-PD1 therapy in mice [32]. Further subtyping of T cells in tumors using single-cell sequencing would uncover whether SCPs may impact ICIs through the modulation of T cell stemness. In addition to T cells, other immune cells such as macrophages [33] and dendritic cells [34] also contribute to the overall outcome of ICI therapy. How SCPs affect these types of immune cells and whether they are involved in the potentiating effect of SCPs are intriguing questions necessitating further exploration.

Many polysaccharides are barely digestible in the small intestine but can be effectively utilized by microbes in the colon, making them ideal substrates for the gut microbiome. For example, pectin has been reported to enhance anti-PD1 efficacy in a colorectal cancer model [35]. Inulin could greatly enhance the anti-tumor activity of anti-PD1 through reconstructing the microbiota in mice [36]. Similarly, SCPs reshaped the gut microbiota and enriched certain bacteria, indicating its potential as a prebiotic adjuvant to anti-PD1 immunotherapy. In comparison to polysaccharides from plant sources such as pectin, inulin, or other herbs (such as ginseng), SCPs contain abundant sulfate groups in their chemical structures, which may partly contribute to their unique capacity to enrich certain microbial taxa that have been identified to be associated with an improved response to ICI therapy (such as *Lactobacillus gasseri*, *Lactobacillus reuteri*, and *Bifidobacterium pseudolongum*). This renders it a more potentially useful dietary adjuvant that could enhance the efficacy of immunotherapy. This shift in the microbiome profile influences the efficacy of immunotherapies by interacting with the host’s immune system. Importantly, the fecal microbiota transplantation (FMT) experiment further validated the critical role of the gut microbiota in this synergy. Mice that received SCP-treated animal microbiota showed a high response against PD1 therapy with improved anti-tumor immunity, further supporting the hypothesis that the immunomodulatory effects of SCPs are at least partially mediated by changes in the gut microbiota. This finding is consistent with previous studies that have demonstrated the ability of certain gut microbes and their metabolites to enhance anti-tumor immune responses, particularly by modulating T cell activity.

In addition to cell components, metabolites produced by the gut microbiota have been reported to influence the response to ICI therapy. Inosine, a bacterial metabolite that can be produced by *Bifidobacterium pseudolongum*, could activate Th1 through adenosine receptor signaling and activate effector T cells [37]. Another intestinal metabolite, Trimetlylamine-N-oxide (TMAO), which is produced from a choline-rich diet by certain gut microbes, enhanced the ICI activity in triple-negative breast cancer by inducing pyroptosis in tumor cells and elevating CD8^+^ T cell-mediated anti-tumor immunity [38]. It has been reported that secondary metabolites of mulberry leaves inhibit the development and progression of lung cancer by targeting the PD-L1/PD-1 signaling pathway and enhancing T cell-mediated immune responses [39]. A particularly compelling aspect of our study was the identification of the gut microbiome as a key mediator of observed synergies between SCP and anti-PD1 therapies. Metagenomic and metabolomic analyses showed that SCP supplementation altered the composition of the gut microbiota and increased the abundance of specific metabolites, such as indole-3-carboxylic acids, which has been reported to decrease regulatory T cells by inhibiting indoleamine 2,3-dioxygenase (IDO1) expression and competing with Kyn for binding to the aryl hydrocarbon receptor [40]. It is possible that the changes in immune cell profiles in SCP-treated mice are induced by this metabolite, which merits further exploration.

## 5. Conclusions

In conclusion, it is demonstrated that SCPs effectively work in synergy with anti-PD1 to reduce the MC-38 tumor burden in mice. These boosting effects were associated with an increase in cytotoxic CD8^+^ T cells and a decrease in regulatory CD4^+^ T cells. Moreover, reshaped microbiota at least partly mediated the beneficial effect of SCPs. The limitation of this study lies in the fact that although the role of microbiota in mediating the beneficial effect of SCPs has been analyzed, how SCPs modulate these specific bacteria and the molecular pathways through which those metabolites interact with specific immune cells have not been explored. This is necessary for the future development of related products. Moreover, safety assessment and human clinical trials are still warranted before the application of SCPs as a dietary adjuvant in immunotherapy.

## Data Availability

The original contributions presented in this study are included in the article. Further inquiries can be directed to the corresponding author.
